# Potential Role of Yoga Intervention in the Management of Chronic Non-malignant Pain

**DOI:** 10.1155/2022/5448671

**Published:** 2022-05-28

**Authors:** Shivani Gupta, Surabhi Gautam, Uma Kumar, Taruna Arora, Rima Dada

**Affiliations:** ^1^Laboratory for Molecular Reproduction and Genetics, Department of Anatomy, All India Institute of Medical Sciences, New Delhi, India; ^2^Department of Rheumatology, All India Institute of Medical Sciences, New Delhi, India; ^3^Division of Reproductive Biology, Maternal & Child Health, Indian Council of Medical Research, New Delhi, India

## Abstract

Pain is an unpleasant and upsetting experience. Persistent pain has an impact on an individual's quality of life which causes stress and mood disorders. There are currently no pain-relieving techniques available that can eliminate pain and offer relief without causing any adverse effects. These factors draw attention to traditional treatments like yoga and meditation, which can reduce biological stress and hence increase immunity, as well as alleviate the psychological and emotional suffering produced by pain. Yoga reduces the stress response and the pain cascade via the downregulation of the hypothalamus-pituitary-adrenal (HPA) axis and vagal stimulation. Yoga is a cost-effective growing health practice that, unlike pharmaceuticals, has no side effects and can help patients stay in remission for longer periods of time with fewer relapses. Yoga not only reduces stress and depression severity but also improves functional status and reduces pain perception. This article highlights the impact of yoga on pain management and on a malfunctioning immune system, which leads to improved health, pain reduction, disease management, and improvement in overall quality of life.

## 1. Introduction

Chronic non-malignant pain is defined as pain that lasts longer than three months or pain that lasts longer than the predicted recovery time. It can be associated with trauma or disease or can occur *de novo* [1]. Pain is a distressing sensory and emotional experience. Chronic pain is commonly thought to be caused by a variety of factors, including physical, sociocultural, and psychological deficits [2]. It is a widespread health problem that has negative consequences for both patients and society, including diminished productivity, lower quality of life, and a higher cost to the healthcare system. Multidisciplinary pain programs appear to be the best therapeutic choice for patients with complicated chronic pain [3]. There is, therefore, a need for a well-designed interdisciplinary pain management program, to address the psychological aspect of pain diseases as well such as low back pain, osteoarthritis (OA), rheumatoid arthritis (RA), headache, neck pain, fibromyalgia, and irritable bowel syndrome.

The literature review in this article was done using the advanced search engine in the PubMed database and used a combination of English keywords, i.e., “chronic pain,” “yoga,” and “inflammation.” Pain management is one of the most challenging tasks for medical practitioners. For better pain management, several pain-relieving medications are available like local anesthetics, non-steroidal anti-inflammatory drugs (NSAIDs), weak opioids (tramadol), and strong opioids (morphine) which are usually recommended by physicians for the treatment of moderate to severe pain [4, 5]. All these pain-relieving medications provide pain relief but for a short duration with accompanying side effects like gastrointestinal dysfunction, nausea, vomiting, and even respiratory depression in the case of opioids [6, 7]. The persistence of pain affects the quality of life of an individual simultaneously generating stress and mood disorders. Till now, no such pain-relieving measures are available which can alleviate the pain completely and provide relief without any side effects. These lead to the attention towards traditional therapies such as yoga and meditation which can minimize biological stress and correspondingly improve immunity and can also counter the psychological and emotional suffering caused due to pain.

Pain is an unpleasant experience which carries both sensory and emotional aspects that are linked with potential or actual tissue damage. Pain arises due to tissue injury and repetitive noxious stimuli which leads to unpleasant sensation. This sensation is processed by complex and integrative signaling mechanisms from the periphery to the central nervous system (CNS) where inputs are ultimately integrated and then a reflex response is generated [8]. This mechanism includes ascending pathway and descending pain pathway. This neural pathway consists of three relay stations. At the periphery, the free nerve endings are called nociceptors which belong to unmyelinated C-fibers, responsible for dull pain, and myelinated A*δ* fibers which carry acute sharp pain [9, 10]. C-fibers have a slow conduction velocity of 2 *μ*m/second and the diameter of nerves is less than 2 *μ*m. However, A*δ* fibers are the smallest myelinated nerves and have a fast conduction velocity of 3 m/sec. The diameter of the nerves is about 2–5 *μ*m [9]. These are primary afferent fibers whose cell bodies are located in the dorsal root ganglion. These nerve endings have specific receptors for different stimuli such as Transient receptor potential vanilloid (TRPV) for temperature, P2X receptors for purinergic receptors for adenosine triphosphate (ATP), acid-sensing ion channels for hydrogen ions, or polymodal receptors that can be activated via different types of stimulus [11].

When tissue damage occurs due to a noxious stimulus, it leads to the production of inflammatory mediators such as prostaglandins which sensitize the nociceptors to accepting the stimulus or directly activate them. Furthermore, other molecules like histamine, bradykinin, nerve growth factor, tumor necrosis factor (TNF), interleukins (ILs), ATP, hydrogen ions, and potassium are released by infiltrating inflammatory cells which directly activate the nociceptors [12]. Activation of nociceptors causes the opening of voltage-gated ion channels accompanied by the influx of ions such as sodium and calcium into the cytoplasm to generate an action potential. These signals are transmitted to the primary sensory neurons of the dorsal root ganglion (DRG) followed by transmission to secondary sensory neurons located in the dorsal horn of the spinal cord (Rexed lamina I, II, and V) [8]. The fibers of spinal neurons decussate and ascend to form a synapse with the tertiary sensory neuron located in the ventroposterolateral nucleus of the thalamus. Finally, the tertiary neuron fibers send signals to the somatosensory cortex where pain signals are perceived. During their course of transmission to the brainstem region, collateral fibers also send signals to the reticular system which is responsible for arousal during pain. The descending pathway includes the periaqueductal grey (PAG) region which receives inputs from the anterior cingulated cortex, hypothalamus, amygdala, and frontal lobe and also receives the ascending input from the spinal dorsal horn. Then, PAG integrates the information and sends it to RVM (including nucleus raphe magnus and nucleus reticularis gigantocellularis) according to which the processing of nociceptive signals is regulated by the spinal dorsal horn neurons [8, 13]. The cannabinoid and opioid systems and the neurotransmitters like norepinephrine and serotonin are involved in regulating the facilitatory and inhibitory mechanism of the pain pathway.

## 2. Modulation of Pain Signal Transmission via Gate Control Theory

The theory was reported by Melzack and Wall in 1965. The concept suggests that inhibition or decrease in transmission of pain sensation can be possible through activation of non-painful sensation [14]. The process involves peripheral pain-carrying fibers (A*δ* & C-fibers) which communicate with the second-order neuron located in substantia gelatinosa (Laminae II) of the dorsal horn in the spinal cord. The large fibers carry non-painful input (such as A*β* fibers) which communicate with inhibitory interneurons of laminae II and also make synapses with primary afferent fibers of pain and second neurons. So, the non-painful input carrying fibers activate the inhibitory interneuron, leading to the release of gamma-aminobutyric acid (GABA), resulting in presynaptic inhibition of noxious stimulus.

Based on gate control theory, some stimuli are used to generate non-noxious signals for closing the gate for pain signals such as transcutaneous electrical nerve stimulation (TENS), massage, heat and cold therapy, and movements of joints [14–16]. The gate control theory mainly suggests that how pain signals are perceived by the brain through the body's nervous system can be inhibited or decreased and enhanced by psychological factors [17]. So, it is suggested that mind-body practice like yoga can inhibit pain signals and it has the potential to change the way an individual experiences pain.

Pain can be classified based on duration, site of origin, or location. Following are few types of pains which can be encountered upon various external and internal noxious stimuli.

### 2.1. Acute and Chronic Pain

When pain lasts for hours to days or for short period then it is considered acute pain. Acute pain mainly acts as a warning signal for the body towards the harmful stimuli. It is specific, sharp, and usually comes suddenly.

Acute pain disappears when the cause of pain goes away (postoperative pain, fracture of bones, cuts, and wounds). However, chronic pain lasts for more than six months and it persists even after the healing and removal of the cause of pain (arthritis, fibromyalgia, cancer, and neuropathic pain) [18]. Chronic pain also produces emotional stress, anxiety, and depression which adversely affect the quality of life of an individual [19].

Based on mechanism and function, pain can be classified as nociceptive, inflammatory, and neuropathic.

### 2.2. Nociceptive Pain

Nociceptive pain occurs due to internal tissue damage when expose to noxious stimuli like extreme heat, cold, or trauma. The nerve endings which are activated by such stimuli are nociceptors which are located in the skin, bone, muscles, joint capsules, and other tissues. They sense chemical, mechanical, and thermal sensations. The nociceptors are A*δ* myelinated fibers, activated by thermal or mechanical stimuli. However, unmyelinated C-fibers are activated not only by thermal and mechanical but also by chemical stimuli [8].

### 2.3. Neuropathic Pain

Neuropathic pain is defined as the occurrence of pain due to a lesion or disease of somatosensory nerves. It mainly arises from the pathology of the nervous tissue due to injury, diseases such as diabetes, nerve compression, and autoimmune diseases. This causes inflammation within the nerve tissue that could lead to both peripheral and central sensitization. Peripheral neuropathy showed alteration in electrical properties of sensory nerves, resulting in impairment of excitatory and inhibitory signaling [18]. In turn, sensory signals transmission and disinhibition mechanism are altered at the spinal level which could move to the state of hyper excitability. With time this sequence of changes from the periphery to the CNS contributes to neuropathic pain becoming chronic. Alteration in the ion channels, within the affected nerves for example upregulation of sodium channels lead to increased excitability, signal transduction, and increased release of neurotransmitters at the spinal cord terminus of the primary afferent fibers [20, 21]. Enhanced activation of spinal neurons expands the field of many modalities (low threshold A*β* and A*δ* fibers). This causes the increase in excitation of second-order neurons in the spinal cord which generates central sensitization. The increase in signals further increases the excitatory release of pain facilitatory transmitters that phosphorylate the N-methyl-D-aspartate (NMDA) and *α*-amino-3-hydroxy-5-methyl-4-isoxazole propionic acid (AMPA) receptors, contributing to the state of hypersensitivity [18]. Moreover, the non-neuronal cells such as astrocytes and microglia also contribute either directly or indirectly to the development of hypersensitization [22].

### 2.4. Inflammatory Pain

Pain caused by activation of nociceptors via the release of inflammatory mediators by damaged cells is known as inflammatory pain. Inflammation occurs as a natural biological response produced by the body tissues as a reaction to noxious stimuli to remove the necrotic cells and initiate the tissue repairing process. During inflammation, neutrophils move through the bloodstream to the injured site followed by the release of chemical mediators. Other responses like the release of platelet-activating factor (PAF) by mast cells followed by the release of serotonin (5-HT), and macrophages augment the inflammatory response [23]. The inflammatory exudate is rich in nerve growth factors, cytokines, protons, and ions. Together, it produces cardinal inflammatory signs such as redness, swelling, hotness, and inflammation at the site due to increased blood flow. These mediators like bradykinin, prostaglandins, and 5-HT can directly activate their receptors and G-protein receptors present at the peripheral nerve ending [8]. This is accompanied by activation of a cascade of signaling mechanisms such as protein kinase A and C signaling pathway which further recruits more receptors and enhanced the sensitivity of primary afferent fibers resulting in peripheral sensitization [24]. As a result, non-noxious stimuli like light touch produce pain sensation (allodynia) or normal painful stimuli produce intense pain sensation (hyperalgesia) [25].

Even inflammatory pain can be categorized into two types: acute and chronic. As a first respondent to the harmful stimuli, accumulation of leukocytes and plasma cells at the site of injury takes place to assist the inflammatory process. This stimulates the activation of primary afferent fibers which are normally mediated by A*δ* fibers, resulting in the generation of intense pain which lasts for a short duration called acute inflammatory pain. However, in chronic inflammatory pain, prolonged inflammation is accompanied by the persistence of pain which lasts beyond the healing period. The nociceptive signals are typically mediated by C-fibers and they occur due to the recruitment of mononuclear cells at the inflammatory site as well [8]. Some other mediators are also released from the necrotic tissue during the inflammatory process like 5-HT, kinins, histamine, nerve growth factors (NGF), ATP, glutamate, leukotrienes, nitric oxide (NO), NE, and protons which also contribute to inactivation of nociceptors within the inflamed area [26]. Further, this increases the afferents' input in the dorsal horn of the spinal cord and leads to the development of central sensitization resulting in persistent pain.

### 2.5. Biomolecules Involved in Induction, Transmission, and Modulation of Pain Signaling

Glutamate is the main neurotransmitter involved in the shaping of transmission of pain signaling. It is also involved in the generation and maintenance of central sensitization [27, 28]. Substance P, calcitonin gene-related peptide (CGRP), and ATP are also released by primary sensory neurons which act as a transmitter and neuromodulators and they are distributed both at the spinal cord and peripheral nerve endings. Stimulation of primary sensory fibers releases these transmitters to bind to neurokinin-1, NMDA, and AMPA, CGRP receptors on the postsynaptic site in the dorsal horn and also bind to the receptors present on the microglia and astrocytes. Microglia release pro-inflammatory mediators which act in a paracrine manner and enhanced the pain input at the dorsal horn level in the spinal cord. The inflammatory mediators like TNF, IL-1, and IL-6 play important role in the induction and maintenance of pain signals. Activation of chemokines receptors, present on the peripheral nociceptors, lowers the threshold of DRG neurons and leads to the state of hypersensitivity. Transient receptor potential vanilloid 1 (TRP) or capsaicin-sensitive receptors are well known in the pain signaling mechanism [29]. TRPV1 is widely distributed in the unmyelinated C-type sensory nerve fibers and partially in the myelinated A*δ*-type sensory nerve fibers CNS. It activates by chemical and noxious heat stimuli in the peripheral and CNS [30–32]. Activation of TRPV1 increases the Ca ion influx and release of pain facilitatory neurotransmitters and neuropeptides into the dorsal horn of the spinal cord [33–35]. TRPV1 also play a role in the activation of spinal glial cells and contributes to the induction and maintenance of pain [36]. Multiple phosphorylation sites are present on TRPV1 through which various intracellular signaling cascade is activated such as protein kinase A (PKA), protein kinase C, Ca^2+^/calmodulin-dependent kinase II (CaMKII), and Ca^2+^-dependent phosphatase calcineurin which contribute in hyperalgesia or sensitization of primary afferents to mechanical and thermal stimuli [37]. Besides these, oxidative stress plays role in the induction and maintenance of neuropathic and inflammatory pain [38–40]. Reactive oxygen species contribute to neuropathic pain by triggering the phosphorylation of CAMKII signaling [39].

Pain and inflammation are closely related and integrated, not merely via acute peripheral sensitization at the joint but also derived from the modulation in the processing of pain signals at the central level. RA is an inflammatory joint disease that occurs with a prevalence rate ranging from 0.3–4.2%, based on the studied population [41, 42]. RA pain mainly occurs in small joints such as joints of the hand and foot, wrist, ankle, knee, and hip joints.

For the management of pain symptoms, a variety of treatment options have been explored to help patients manage their symptoms of pain, including pharmacological approaches, physical therapy, exercise, surgery, psychological therapy, and complementary and alternative therapies. Pain medications, such as opioids, are frequently given. The use of opioids in the treatment of chronic non-malignant pain is a contentious topic because of the adverse effects, short-term efficacy, and safety of pain medications, as well as the danger of abuse and addiction [43]. Various adverse effects of opioids have been listed in Table 1 [44]. Patients with chronic non-malignant pain face a complicated interaction of biological, psychological, and social variables, thus therapy must take all of these elements into account [45]. Psychological factors play a significant role in deciding whether or not a patient with chronic non-malignant pain will respond to therapy [46].

## 3. Role of Tryptophan Metabolites Formed via Kynurenine Pathway in Pain

Tryptophan is an amino acid that metabolizes to form various active biological molecules such as serotonin, NAD^+^, and melatonin. The metabolism of TRP involves mainly two routes: the serotonin pathway which includes 5% TRP metabolism and 95% is taken place by the KP pathway resulting in the formation of kynurenines. Kynurenine is involved in the regulation of several biological processes such as host-microbiome signaling, the excitability of neurons, and the response of the immune cells [47]. In the KP pathway, TRP converts to N-formyl-L-kynurenine via three enzymes: indole amine 2,3-dioxygenase 1 and 2 (IDO 1 and (2) and tryptophan 2,3-dioxygenase (TDO) and rapidly converted to L-kyukynurenineYN). The downstream cascade of the L-kynurenine can undergo three transformations, also called kynurenines: 3-hydroxykynurenine (3-HK), 3-hydroxyanthranilic acid (3-HAA), and then quinolinic acid (QUIN) is formed via enzymatic action of kynurenine 3-monooxygenase (KMO) and anthranilic acid (AA). The quinolinic acid (QUIN) acts as a precursor for NAD^+^ synthesis.

**Figure 1:**
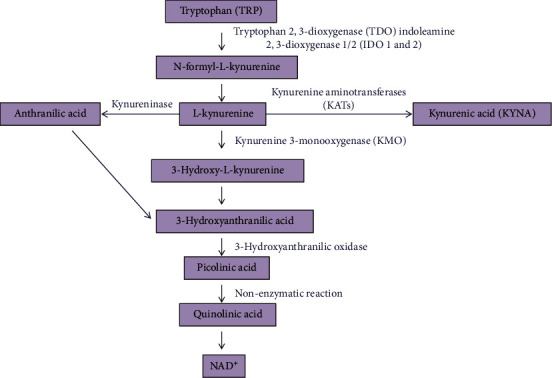
Illustration of the metabolism of tryptophan via the kynurenine pathway.

Glutamate is the predominant excitatory neurotransmitter in the nervous system. Also, KYN is converted into kynurenic acid (KYNA) through irreversible transformation via kynurenine aminotransferase (KAT) enzymes, the end product of its lateral branch. Glutamate binds to AMPA and NMDA receptors which regulate pain transmission and cognitive function. QUIN is an agonist of NMDA receptors leading to a neurotoxic effect. The KYNA acts as an agonist for the NMDA receptor but plays a dual role on AMPA receptors. At lower concentrations, it facilitates AMPA receptor response while at higher concentrations it antagonizes the AMPA receptors' action by allosteric modulation of desensitization of AMPA receptors [48]. In vitro studies suggested that astrocytes are mainly involved in the synthesis of neuroprotective KYNA, and activation of microglia/macrophages during various neuroimmunological diseases leads to the synthesis of neurotoxic QUIN [49, 50]. The link between the development of neuropathic pain and the activity of Kynurenine pathway enzymes was explored in different neuropathic pain models. Several studies reported the upregulation of KP enzymes (IDO 1/2, KMO, and KYNU) in the CNS tissues in various neuropathic pain models [51, 52] (Figure 1). Moreover, inhibition of IDO 2 and KMO enzymes resulted in alleviation of mechanical, tactile, or thermal hypersensitivity in neuropathic pain models [53].

RA is the most common inflammatory arthritis. The role of yoga as an effective adjunct intervention has been documented in the literature to assist in the management of chronic diseases like RA concerning its clinical symptoms like pain perception, stress management, disability outcomes, sleep quality, functional ability, QOL, and psychosocial outcomes [54–56]. RA has a multifactorial etiology, diverse pathogenesis, heterogeneous clinical phenotypes and, this disease comprises a psychosomatic component, hence an integrative approach of yoga might improve clinical outcomes in RA by bringing changes in all interconnected biological components and at various levels-molecular, cellular, organ systems, and the person as a whole. RA serves as a model for chronic inflammatory pain. With this context in mind, this article contains RA as an inflammatory model and the aim is to investigate the effects of yoga and its mechanism of action and analyze their association with the clinical health outcomes including disease activity, functional status, depression severity, and quality of life.

### 3.1. Mechanism of Pain in RA

**Figure 2:**
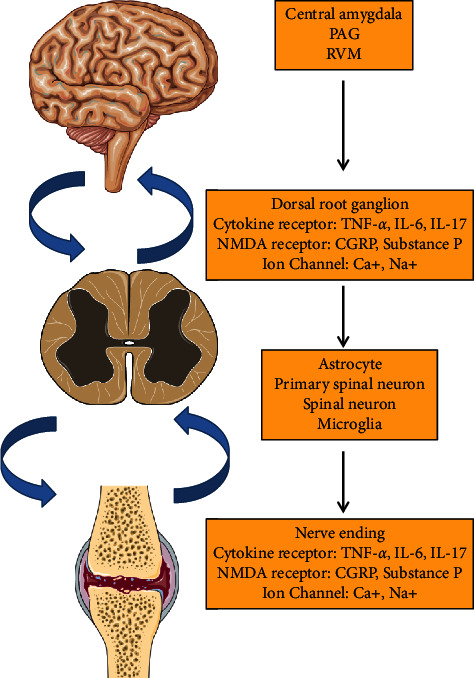
Schematic diagram showing pain mechanism in RA (modified from Cao et al., [59]).

RA is associated with inflammation of the synovial that correlates with the severity of pain in joints. RA pain arises as a result of the combined interaction of joint pathogenesis and modulation in the neuronal sensitivity throughout the nociceptive pathway either at the peripheral or the central level (both spinal cord and brain) [42, 57]. Inflamed synovium is the manifestation of the release of pro-inflammatory mediators by local immune cells. This causes direct activation and sensitization of sensory-free nerve endings called nociceptors, present not only on the synovium but also in the joint capsule, the outer region of menisci, ligaments, muscles, subchondral bone, and tendon sheath [57, 58]. Even during the late phase, the osteochondral junction is disrupted by subchondral erosion and exposes the nerve ending which approaches the degenerative part resulting in an enhancement of inflammatory response. These free nerve endings are the peripheral process of primary sensory neurons located in the dorsal root ganglion and carry pain signals to secondary sensory neurons located in the spinal cord [8, 58]. Direct stimulation induces pain and sensitization; as a consequence normal pain can be felt due to non-noxious stimuli (allodynia) such as pressure, weight-bearing, and movement [57]. The following illustration shows the central and peripheral mechanisms of pain and the various components involved in the process (Figure 2).

### 3.2. Mechanism of Peripheral Sensitization in RA

Peripheral sensitization leads to the genesis of chronic pain. It constitutes a decrease in the threshold of nociceptors and an increase in their response towards the stimulus which occurs because of post-translational changes in and alteration in the trafficking of ion channels and transducer receptors. This is initiated by the activation of nociceptors by local inflammatory mediators at the inflammation site, resulting in a state of pain hypersensitivity [60, 61]. This is known as primary hyperalgesia. Medications that bind to opioid receptors are increasingly being prescribed for the treatment of multiple and diverse chronic painful conditions. Their use for acute pain or terminal pain is well accepted. Their role in the long-term treatment of chronic non-cancer pain is, however, controversial for many reasons. One of the primary reasons is the well-known phenomenon of psychological addiction that can occur with the use of these medications. Abuse and diversion of these medications is a growing problem as the availability of these medications increases and this public health issue confounds their clinical utility. Also, the extent of their efficacy in the treatment of pain when utilized on a chronic basis has not been definitively proven. Lastly, the role of opioids in the treatment of chronic pain is also influenced by the fact that these potent analgesics are associated with a significant number of side effects and complications. It is these phenomena that are the focus of this review.

Common side effects of opioid administration include sedation, dizziness, nausea, vomiting, constipation, physical dependence, tolerance, and respiratory depression. Physical dependence and addiction are clinical concerns that may prevent proper prescribing and in turn inadequate pain management. Less common side effects may include delayed gastric emptying, hyperalgesia, immunologic and hormonal dysfunction, muscle rigidity, and myoclonus. The most common side effects of opioid usage are constipation (which has a very high incidence) and nausea. These side effects can be difficult to manage and frequent tolerance to them does not develop; this is especially true for constipation. They may be severe enough to require opioid discontinuation and contribute to under-dosing and inadequate analgesia. Several clinical trials are underway to identify adjunct therapies that may mitigate these side effects. Switching opioids and/or routes of administration may also provide benefits for patients. Proper patient screening, education, and preemptive treatment of potential side effects may aid in maximizing effectiveness while reducing the severity of side effects and adverse events. Opioids can be considered broad-spectrum analgesic agents, affecting a wide number of organ systems and influencing a large number of body functions [44]. The signaling molecules are involved in mediating peripheral sensitization such as protons, ATP, prostaglandins (PGE2), leukotrienes, nerve growth factor (NGF), cytokines (IL-6, IL-1*β*, TNF-*α*), chemokines, neuropeptides, CGRP, substance P, bradykinin, histamine, lipids, and diverse proteases [62]. Inflamed synovium manifests an increased level of prostaglandins and bradykinin in RA patients that can directly activate unmyelinated sensory nerves [58]. Furthermore, NGF-*β* and cytokines such as IL-1, IL-6, and TNF-*α* also increased which can sensitize the peripheral sensory nerves in synovium and subchondral bone of RA patients [60, 63]. The stimulation of these nociceptive fibers activates the intracellular signaling pathway following the activation of the phosphorylation cascade in the neuronal cell. Synovitis is also associated with alteration in the expression of neurotransmitters, neuromodulators, and their receptors at nociceptive neurons located in the DRG and dorsal horn of the spinal cord [60].

### 3.3. Mechanism of Central Sensitization in RA

Central sensitization is responsible for a variety of chronic pain diseases including RA [64, 65]. Central sensitization is the enhanced response of nociceptive neurons in the CNS towards their normal and subthreshold afferent inputs. This is known as secondary hyperalgesia. Stimulation of nociceptors results in the generation of action potential leading to the release of excitatory neurotransmitters at the spinal level. At the spinal level, central sensitization occurs in the dorsal horn which relieves inputs from an enlarged receptive field leading to pain hypersensitivity. This occurs in two steps: in the acute stage, primary afferent fibers released glutamate which binds to their receptors on postsynaptic neurons and the chronic stage includes activation of spinal microglia and the transcription of pain regulatory molecules [27]. Glutamate is the key transmitter that activates both NMDA and non-NMDA receptors on spinal cord neurons [63]. This leads to the release of various inflammatory mediators and in turn, initiates the central hypersensitivity or central sensitization [57, 66–68]. Synovitis is accompanied by upregulation of SP, CGRP, and their receptors in the spinal cord [27, 57]. Stimulation of primary sensory fibers releases these transmitters to bind to neurokinin-1, NMDA, AMPA, and CGRP receptors on the postsynaptic site in the dorsal horn and they also bind to the receptors present on the microglia and astrocytes [69]. Microglia release pro-inflammatory mediators which act in a paracrine manner and enhanced the pain input at the dorsal horn level in the spinal cord [70]. The inflammatory mediators like TNF, IL-1, and IL6 play important role in the induction and maintenance of pain signals [12]. Microglia also secrete other pro-inflammatory molecules like nitric oxide and superoxide anion which act in a positive feedback manner and lead to hyperalgesia [57]. On the other side, astrocytes activation further leads to the recruitment of pro-inflammatory mediators like TNF-*α* which modulates pain signaling in various ways. TNF-*α* can directly act on neurons and modulate synaptic activity. Activation of astrocytes by TNF-*α* induces phosphorylation of pJNK1, mitogen-activated protein kinase (MAPK) which regulates the gene transcription. Pain signals from sensory neurons of the dorsal horn in the spinal cord transmit to the sensory neurons of the ventroposterolateral region of the thalamus. Then to the somatosensory cortex where finally pain signals are perceived in the brain. In response, the pain regulatory mechanism of the CNS activates the descending pain pathway. These pathways transmit signals from the brain through the brainstem to the spinal cord. In the brainstem, afferent signals are received by periaqueductal grey (PAG) from the frontal cortex, amygdalae, and hypothalamus related to stress and mood which influence the perception of pain. The signals are integrated by PAG and transmitted to the rostral ventromedial medulla (RVM) in the brainstem. Depending on the activation of specific pathways RVM can transmit or inhibit the pain [42]. Even RA pain is also manifested due to non-inflammatory factors like sleep disturbances, anxiety, and depression which affect the perception of pain [71].

RA has become one of the major public health problems and around 1% of the world population is affected [72]. Among these, it is more common in women than men. The condition of RA is manifested by pain, swelling, stiffness, loss of joint function, and increased acute-phase reactant levels [73]. Management of RA includes Disease-Modifying Anti-rheumatic Drugs (DMARDs), Non-steroidal anti-inflammatory drugs (NSAIDs), Glucocorticoids (GC), and even opioids [74]. However, this provides pain relief but never cures it completely so it is necessary to search for effective therapy which includes better pain management with fewer or no side effects. Individuals suffering from chronic pain may benefit from mindfulness training as a therapy option [75, 76]. The deliberate and nonjudgmental conscious awareness of the current moment is characterized as mindfulness. Psychological factors have been proven to have a significant influence on how patients feel and tolerate pain, yet they are frequently overlooked when chronic non-malignant pain treatment strategies are adopted. Chronic pain is associated with dysregulation of emotional and cognitive functions leading to anxiety, depression, altered attention, fear, etc. [2]. These alterations are also linked to the phenomena of “pain catastrophizing,” which is described as the repeated negative thoughts that occur during or before pain [77]. Anxiety, depression, drug use disorders, and sleep problems are just a few of the variables that can make the pain worse. Hence, management of these comorbidities becomes essential to control the overall patient's pain state. A multi-disciplinary treatment approach model which incorporates both pharmacological and non-pharmacological interventions is more effective in managing chronic pain than single treatment modalities [78]. A systematic review reported that there was evidence of better effectiveness of multi-disciplinary treatment groups compared to the single treatment group in the management of musculoskeletal pain [78]. There is a wide range of social, psychological, non-pharmacological, and non-opioid pharmacological treatment options available for patients with chronic non-malignant pain. It may be essential to test several therapies combinations to discover the optimal treatment option for each patient [43]. There are various non-pharmacological treatment options in the management of chronic pain like physical therapy, yoga, tai-chi, massage, acupuncture, and cognitive behavioral therapy.

### 3.4. Yoga in the Management of Chronic Pain

**Figure 3:**
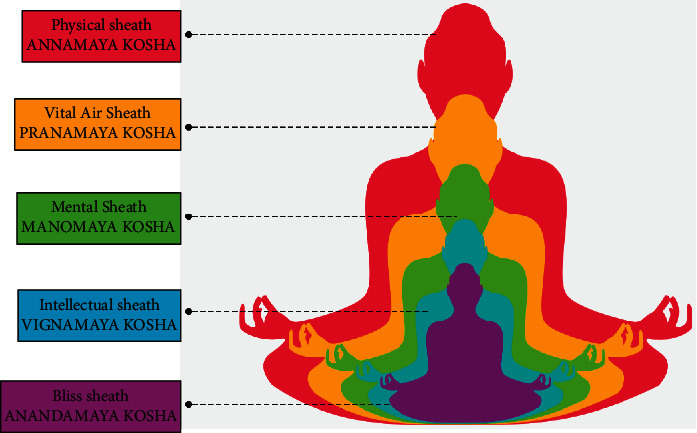
The Panchamaya model (modified from an illustration by Maya Chastain).

As chronic pain is multidimensional and affects an individual's different organ systems, hence an integrated mind body medicine approach like yoga is highly beneficial. Pain is divided into five categories based on its mechanism: peripheral neuropathy, central sensitization, sympathetically sustained pain, nociceptive, and cognitive-affective pain. Every occurrence of pain is evaluated from many perspectives, i.e., sensory, emotional, behavioral, and cognitive [19]. The brain is built to anticipate the worst and tends to take caution by eliciting a strong pain response quickly. For the sake of efficiency, the brain creates streamlined neuropathways based on previous experience for coping with comparable dangers and uses them for each episode of pain. The physical body, physiology, mental appraisal, emotional reaction, and general attitude towards life must all be considered while managing chronic pain [79]. Yoga is a collection of physical, mental, and spiritual disciplines that originated in ancient India and are aimed at controlling the mind as well as recognizing the detached consciousness. The five koshas (Panchamaya model) consist of five main layers of our systems: physical structure, physiological processes, the content of minds, ideas, and attitudes towards our surroundings, and our sense of connection to other people, society, and the Universe [80, 81]. Each of those layers includes tools established by the yoga tradition to promote balance and healing (Figure 3). Yoga is a profound science-based health discipline and can be used as a complementary, integrative, and adjunct medical therapy that impacts the body, and mind as a whole and results in promoting physical and mental health and improving quality of life [82]. Yoga is a technique associated with mind-body relaxation and acts as a cushion to the changing cellular immunity related to the stress. Yoga mainly focuses on regulated breathing practices, mind, and body which includes mild to moderate postural exercises (asana), breathing exercise (pranayama), and meditation (dhyana). Regular practice of yoga is effective in reducing pro-inflammatory biomarkers such as IL-6 and TNF-*α* from their basal level in healthy yoga practitioners [83]. Chronic elevation in these markers leads dysregulated immune response and sets stage for autoimmune progression of inflammatory diseases like RA.

Yoga decreases systemic and local inflammation in RA by normalizing circulation levels (IL-6, IL-17A, and TNF-*α*) and mRNA transcript levels of pro-inflammatory cytokines (IL-6, TNF-*α*) [84]. Yoga also improves autonomic reflex regulation systems and restores the balance between sympathetic and parasympathetic limbs in inflammatory situations [85]. Yoga practice also reduces the level of oxidative stress and improves the anti-oxidant levels [86]. Therefore, one of the possible mechanisms by which yoga could be reducing RA pain is by decreasing the level of inflammatory mediators. This leads to peripheral reduction of inflammatory mediators levels which results in the decrease of stimulation of nociceptors so the transduction and transmission of pain signals are reduced from the periphery to the spinal cord. There is a decrease in the level of oxidative stress at the spinal and brain level due to less activation of spinal neurons and non-neuronal cells. Together, overall it reduces the level of pain hypersensitivity throughout the pain pathway from the periphery to the central level. In addition, the pain pathway is also regulated by GABA, an inhibitory neurotransmitter. In chronic pain, the level of GABA is reduced in the brain and it is also involved in the gate control mechanism of pain signals. This includes the GABAergic interneuron that controls the transmission of signals. Practicing yoga on regular basis also increases the level of GABA which could facilitate the inhibitory action on the transmission of pain signaling.

Yoga is now being used more often to alleviate painful ailments and enhance emotional resilience and enhance threshold to pain. Studies are being conducted to look at probable neuroanatomical changes as a result of yoga practice. A study conducted on North-American yogis analyzed the anatomical alterations in the grey and white matter of the brain which indicated that yogis had more left intrainsular white matter integrity than controls, and they were able to endure more pain than the control group due to their parasympathetic activation and enhanced awareness [87]. Yoga, as an integrated health technique, has both psychological and physical components, making it appropriate as an adjunct management approach for severe, debilitating autoimmune arthritis such as RA [85, 88, 89]. Yoga has a substantial impact on RA because it helps to reduce articular and extra-articular symptoms while also improving systemic indicators of inflammation, oxidative stress, and cellular health [84]. Yoga works through psycho-neuro-immunological systems to attain life's homeostatic equilibrium [90]. Yoga enhanced the quality of life in active RA patients by lowering pain perception, disability quotient, disease activity, and severity of comorbid depression [84, 85, 90–92].

**Figure 4:**
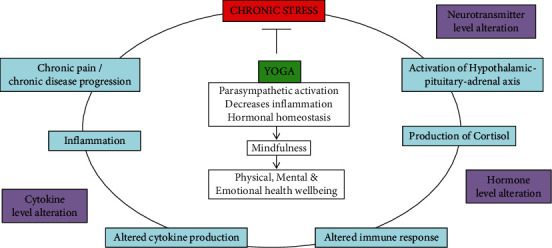
Yoga in reducing chronic stress and its consequences.

Pranayama is a voluntary breathing regulation that is commonly practiced in conjunction with yoga asanas and meditation. The three phases of pranayama consist of the inhalation “puraka,” the breath-holding “kumbhaka,” and the exhalation “rechaka.” Stepwise breath regulation influences the autonomic nervous system's control, which has additional favorable effects on the body's organ systems. The parasympathetic nervous system is activated when pranayama breathing is done with the extended breath retention [93]. The mode of action of yoga is vagal stimulation, which improves baroreflex sensitivity and lowers inflammatory cytokines, and parasympathetic activation, which is linked to anti-stress processes (Figure 4). Yoga lowers stress perception and hypothalamus pituitary adrenal (HPA) axis activation, resulting in improved metabolic and psychological profiles [94].

Yoga is a profound science-based mind body practice that may be utilized as adjunct to medical therapy that affects the whole body and mind, boosting physical and mental health and enhancing the quality of life. It is a low-cost mind-body intervention that, unlike medicines, has no side effects and has a beneficial influence on the entire body, allowing for longer periods of remission with fewer relapses. Yoga has a beneficial influence on the genome and epigenome, resulting in molecular remission and immunological tolerance [85, 95–97]. Various studies from our lab showed that 8 weeks of yoga intervention significantly reduced disease activity, altered methylation patterns, improved mitochondrial integrity and biogenesiss and thus slowed down the rate of functional decline associated with aging, normalized the systemic biomarkers of inflammation, oxidative stress, immune-senescence, aging, and promoted neuroplasticity [90, 91, 98, 99]. This is due to the impact on the genome and epigenome, leading to the normalization of gene expression and epigenetic marks. There was an improvement in various systemic, molecular, epigenetic, and genetic markers associated with RA pathogenesis. Also, there was a favourable clinical outcome of RA with a reduction in disease activity, disability index, pain acuity, depression severity, and improvement in quality of life [90].

### 3.5. Impact of Yoga on Inflammatory Cytokine Profile

Yoga decreases systemic and local inflammation in RA by normalizing circulation levels (IL-6, IL-17A, and TNF-*α*) and mRNA transcript levels of pro-inflammatory cytokines (IL-6, TNF-*α*) [84]. Yoga also improves autonomic reflex regulation systems and restores the balance between sympathetic and parasympathetic limbs in inflammatory situations [85]. Various studies have seen a marked reduction in the levels of inflammatory markers after yoga intervention in various conditions (Table 2).

### 3.6. IL-6

The *IL-6* gene on chromosome 7 encodes a pleiotropic cytokine that plays a crucial role in the etiology of RA and has a significant positive association with disease activity and joint damage [108]. It has pro-inflammatory effects by attaching to transmembrane or soluble receptors and activating gp130, a transmembrane protein, which then triggers the IL-6 signaling cascade. The JAK-STAT signaling pathway stimulates numerous transcriptional factors in response to IL-6 signaling, resulting in CD4^+^*T* cell proliferation, differentiation, and activation [109]. In the presence of transforming growth factor (TGF)-*β*, IL-6 enhances Th17 cell development, which secretes IL-17A, another strong pro-inflammatory cytokine, while inhibiting TGF-*β* induced Treg cell differentiation [110]. As a result, IL-6 disrupts immunological homeostasis by boosting Th17 cell numbers over Treg cell populations. By activating B cells, IL-6 is a powerful stimulator of the HPA axis, causing acute phase responses and the generation of autoantibodies [111]. In inflammatory conditions, the HPA axis is triggered by inflammatory cytokines. Stressors/pro-inflammatory cytokines cause the hypothalamus to release the corticotrophin-releasing hormone, which stimulates the adrenal gland to generate and release cortisol by acting on the anterior pituitary and inducing the production of adrenocorticotropic hormone. Cortisol counteracts the stressor and reduces the prevailing inflammation [112]. However, the HPA axis becomes hypersensitive as a result of persistent inflammatory stimulation and negative feedback regulation of cortisol on the hypothalamus and anterior pituitary begins. This condition leads to adrenal insufficiency. Yoga reduces stress and inflammation by inhibiting the hypersensitive HPA axis and regulating cortisol levels [100].

### 3.7. TNF-*α*

TNF-*α* is one of the most important pro-inflammatory cytokines in the pathogenesis of RA [113]. TNF-*α* functions in increasing vasodilatation and edema formation causing leukocyte adhesion to epithelium through the expression of adhesion molecules and contributing to oxidative stress in sites of inflammation [114]. In the TNF signaling pathway, TNF cytokines interact with its receptor to activate MAPK and NF‐*κ*B signaling pathways resulting in the release of leukocyte inflammatory factors. The released inflammatory factors further activate the membrane receptors leading to a vicious circle of inflammatory responses. It is reported that stimulating the NF‐*κ*B and TNF signaling pathways could promote cell inflammation in RA [115]. Recent studies from our lab showed that yoga aids in the regression of inflammatory processes by the reduction in the levels of TNF-*α* and IL-6 cytokines in RA patients [84, 85].

### 3.8. TGF-*β*

TGF-*β* is another key pleiotropic cytokine that helps maintain immunological homeostasis and produces peripheral tolerance through its regulatory activities. TGF-*β* enhances the differentiation of induced Treg cells using IL-2 and retinoic acid and preserves the survival of naturally existing Treg cells. Treg cells monitor abnormal immune responses and are thought to be protective against an overactive immune system and the onset of autoimmunity [116]. A study from our lab showed that yoga holds the immune-regulatory potential and reduces the severity of RA by elevating *TGF-β* transcript levels and circulating levels of TGF-*β* hence developing immunological tolerance.

### 3.9. IL-17A

IL-17A is responsible for articular symptoms as it induces inflammation in synovial tissue. As ROS damage the mitochondrial DNA, this might be a compensatory strategy to ensure appropriate ATP levels. Simultaneously, there is a reduction in OS and overexpression of genes that preserve mitochondrial integrity, resulting in fewer free radicals being produced. Increased ROS levels cause T helper 17 (Th17) cells to differentiate, resulting in increased production of interleukin IL-17, indicating that mitochondrial changes play a significant role in the Th17 cell effector phenotype. The immune system equilibrium between Tregs and effector Th17 cells is disrupted. [117]. Dysfunction of Tregs fails to maintain peripheral tolerance and results in autoimmunity [118]. TGF-*β*, IL-10, IL-35, and the soluble human leukocyte antigen (HLA)-G molecule are secreted by Tregs, whereas Th17 cells release pro-inflammatory cytokines such as IL-17, TNF-*α*, IL-22, and IL-26, IFN-*γ*, and others [119]. Increased production of inflammatory cytokines by effector Th17 cells and a loss of Treg suppressor activity are both linked to immunological dysregulation in RA [117]. In an unpublished study from our lab, the mean proportion of Th17 cells (CD3^+^CD4^+^IL17^+^RORt^+^*T* cells) in the yoga group decreased significantly from baseline to the eighth week, but the mean percentage of Treg cells (CD3^+^CD4^+^CD25^+^CD127-Foxp3^+^T cells) increased significantly in the yoga group. The mean percentage of aged Th17 cells (CD3^+^CD4^+^IL17^+^ROR*γ*t^+^CD28^−^*T* cells), as well as aged Treg cells (CD3^+^CD4^+^CD25^+^CD127^−^Foxp3^+^CD28^−^*T* cells), has shown a significant overall decline in yoga group as compared to non-yoga group. There was an upregulation of soluble HLA-G levels in RA patients followed by yoga intervention. HLA-G, a non-classical HLA class I molecule, has anti-inflammatory and immune-modulatory effects. It is an excellent reference parameter for autoimmune and inflammatory disease prevention, diagnosis, and therapy since higher levels are linked to less severe illness and fewer relapses in RA patients.

### 3.10. Impact of Yoga on Endogenous Opioids and Neurotransmitters

Encephalin and endorphins are endogenous opioids that are generated largely in the brain and have various effects throughout the body [120]. Opioid antagonists can limit the activity of encephalin and endorphins, which act on opioid receptors. Increased levels of brain-derived neurotrophic factor (BDNF), dehydroepiandrosterone sulfate (DHEAS), and *β*-endorphins suggest a significant improvement in mind-body communication indicators following yoga practice. Yoga raises the levels of BDNF, a key neuroplasticity biomarker, as well as DHEAS, serotonin, neuregulin, and neurotropin [121, 122]. Multiple neurotransmitters in the brain are altered by regular yoga practice. In a study, yoga practice for one hour boosted GABA levels in the brain [123]. BDNF has neuroprotective and neurotrophic properties [122, 124]. Yoga promotes mind-body communication, which controls neuroplasticity and decreases the stress cascade by increasing BDNF, DHEAS, and *β*-endorphins. DHEAS is dysregulated in RA and possesses neuroprotective, anti-oxidant, and anti-inflammatory effects. DHEAS levels were shown to be increased in RA patients when given a TNF antagonist, which enhanced adrenal function [125, 126]. This study also discovered a substantial increase in DHEAS levels after yoga, indicating that yoga can help with emotional control, neurocircuits, cognition, emotional resilience, and memory. Upregulated DHEAS levels following a yoga practice have also been shown to lower depression severity in individuals with major depressive disorder and situations of comorbid depression, such as in RA patients [98].

Another study found that doing yoga for one hour every day for three months reduces adrenocorticotropic hormone (ACTH) and cortisol while increasing serotonin, dopamine, and BDNF in healthy active males [127]. *β*-Endorphins are endogenous morphine produced by the pituitary gland and diffused throughout the body. This neuro-hormone functions as a neuro-regulator and its levels in the blood tend to rise with intense and long-lasting physical exercise [128]. As a result, yoga has the ability to increase *β*-endorphin levels and generate a good sensation of pleasure, wellbeing, and security in RA patients who are already depressed [98]. Yoga has the ability to reorganize the structure and function of the nervous system which was manifested by the upregulation of markers of neuroplasticity. Yoga intervention leads to decline in severity of the disease which further influences psychological health via changes in biomarkers at a systemic level. The yoga group was more compliant with the therapy and able to conduct daily activities without any difficulty as their psychological health and depression severity improved [84]. Improvement in the overall quality of life, as well as health assessment questionnaire, was seen after the yoga intervention, with improvement in the physical, psychological, social, and environment domain scores of the WHOQOL-BREF questionnaire [90]. Yoga improved physical and emotional fitness, psychological health, and overall wellbeing in patients who practiced it [85].

## 4. Conclusion

Yoga, a mind-body medicine and is a profound science and technique for achieving optimal health and well being. It has immune-modulatory properties, lowers pain perception, regulates the psycho-neuro-immune axis, reduces depression severity, reduces disability quotient, and improves the quality of life. Hence, yoga is useful to patients with chronic pain as a complementary treatment that improves physical function and enhances emotional resilience and mental well-being.

## Figures and Tables

**Table 1:** Adverse effects of opioids [44].

S.No.	Organ system	Adverse effects
1	Respiratory system	Respiratory depression, obstructive and central sleep apnea, ataxic breathing, respiratory arrest, and death
2	Central nervous system	Increased risk of falls, cognitive impairment, myoclonus, delirium, depression, somnolence, and sleep disorders
3	Cardiovascular system	Orthostatic hypotension, bradycardia, vasodilation, and an increased risk of cardiovascular events, e.g., myocardial infarction
4	Gastrointestinal system	Constipation, nausea and vomiting, gastric reflux, delayed gastric emptying, abdominal cramping, and distension
5	Immune system	Decreased wound healing, pruritus, altered cytokine production, increased histamine release, inhibition of macrophage, neutrophil, and natural killer cell activity and recruitment, increased HIV replication, and cancer progression
6	Endocrine system	Opioid-induced endocrinopathy (usually only with high opioid doses, long-term), resulting in decreased libido, testicular atrophy, early menopause, and sexual dysfunction

**Table 2:** Reduction of various inflammatory markers in different disease conditions.

Condition	Inflammatory markers	References
Rheumatoid arthritis	IL-6, IL-17A, TNF-*α*, and ESR,	[84]
	CRP, ROS	[91]
	NFKB	[100]
	IL-10	[101]
Type 2 diabetes	IL-8, IL-6, IL-18, CRP, and IL-6	[102]
Low back pain	TNF-*α*	[103]
Depression	IL-6, Cortisol	[98]
	IL-6, TNF-*α*, and CRP	[104]
Multiple sclerosis	IL-1, IL-2, IL-4, IL-6 IL-10, IFN-*γ*, TGF-*β,* and TNF-*α*	[105]
Inflammatory bowel disease	IL-6	[106]
Cancer	NFKB, IL-6, and GP130-JAK pathways	[107]
